# Black Sexual Minority Men’s Experiences in MPowerment Interventions: Implications for HIV Prevention

**DOI:** 10.1007/s12529-024-10275-5

**Published:** 2024-03-18

**Authors:** Rodman E. Turpin, Aaron D. Camp, C.J. Mandell, Rochelle R. Davidson Mhonde, Typhanye V. Dyer, Kenneth H. Mayer, Hongjie Liu, Thomas Coates, Bradley Boekeloo

**Affiliations:** 1 Department of Global and Community Health, College of Public Health, George Mason University, Fairfax, VA, USA; 2 Heller School for Social Policy and Management, Brandeis University, Waltham, MA, USA; 3 INOVA Health System, Fairfax, VA, USA; 4 Department of Epidemiology and Biostatistics, School of Public Health, University of Maryland, College Park, MD, USA; 5 The Fenway Institute, Fenway Health, Boston, MA, USA; 6 Department of Medicine, Beth Israel Deaconess Medical Center, Boston, USA; 7 Harvard Medical School, Boston, MA, USA; 8 David Geffen School of Medicine, University of California, Los Angeles, Los Angeles, CA, USA; 9 Department of Behavioral and Community Health, School of Public Health, University of Maryland, College Park, MD, USA

**Keywords:** PrEP, HIV, Stigma, Racism, Homophobia, Intersectionality, MPowerment

## Abstract

**Background:**

Black sexual minority men (BSMM) are disproportionately vulnerable to HIV acquisition; the MPowerment model is one community-based framework for preventing HIV in this population. It focuses on developing a supportive network of peers to promote health messaging, reduce stigma, and improve resilience. While these interventions have demonstrated general success, there are important challenges related to race, sexuality, and internalized stigma. Our study aimed to explore these experiences among BSMM in MPowerment models focused on HIV prevention.

**Method:**

We conducted 24 qualitative interviews of BSMM attending HIV prevention–related MPowerment events in the greater D.C. Metropolitan area. In-depth interviews were conducted via phone, and interviews were analyzed using thematic analysis.

**Results:**

We identified four themes from the transcript analysis process: Black queer intersectional social support and community, HIV-related information and destigmatization, social status, and sexuality. Within each of these themes, we identified relationships with overall HIV prevention messaging, including barriers to PrEP use. Barriers related to social status were especially prevalent and described as unique to the D.C. metropolitan area.

**Conclusion:**

Overall, MPowerment event spaces provide a forum for BSMM to feel safe and supported while gaining important HIV-related knowledge and prevention access. Challenges related to social status and destigmatization of sexuality are important considerations in designing and implementing this model, especially related to PrEP promotion.

## Introduction

Black sexual minority men (BSMM) are only 1% of the US population but account for 26% of all new HIV diagnoses in the United States [1]. If current trends persist, approximately one in two BSMM are projected to contract HIV in their life-time [1]. Pre-exposure prophylaxis (PrEP) is a particularly effective tool for addressing this, as it reduces HIV incidence by 90 to 98%, though BSMM PrEP use is much lower than that of White or Hispanic/Latino SMM [2–7]. These disparities have far-reaching implications for the HIV epidemic, and are thus an important focus of public health efforts.

Much of these disparities are rooted in social and structural discrimination and stigma, as described by intersectionality [8]. This theoretical framework posits that systems of oppression intersect to create distinct effects on members of multiple marginalized identities, resulting in greater levels of stigma and discrimination beyond the combined experiences of their individual identities [8, 9]. For example, BSMM experience racism, homophobia, and a unique intersection of the two that is not experienced by Black heterosexual men or sexual minority men of other racial groups [9]. This may include racism, homophobia, and related socioeconomic barriers to HIV prevention services that disproportionately affect BSMM, including poverty and homelessness [3, 10]. Experiences of racism and homophobia may be internalized, resulting in greater distrust of medical services and stigmatization of HIV prevention utilization; this creates a direct barrier to HIV prevention [2, 11–13]. To address these challenges, interventions have increasingly focused on addressing multi-level social and community factors to promote HIV prevention among BSMM. Several economic, social, and community-level factors, such as poverty, racism, homophobia, and related stigmas, are potential intervention targets among SMM [3, 14–17]. Inversely, psychosocial interventions that include peer-based engagement have a positive impact on HIV risk reduction outcomes in several populations of SMM [5, 6, 14, 15]. Several studies, including interventions utilizing BSMM community networks to decrease HIV incidence, have explored social contexts that affect HIV prevention among SMM. [5, 6, 14, 15]

The MPowerment model is one community-based framework for preventing HIV among SMM [18, 19]. This intervention model was developed by Kegeles, Hayes, and Coates to reduce sexual risk behavior among young gay and bisexual men in a midsized Oregon community [18]. The model focuses on developing a supportive network of SMM, allowing for effective peer-to-peer health messaging on topics such as behavioral and sexual health [18]. The model’s theoretical basis is Diffusion of Innovations; this framework explains how an idea gains momentum within a social network and diffuses through members of that network [18, 19]. SMM, including BSMM, may adopt new behaviors from members of their networks. As part of the MPowerment model, BSMM “innovators” lead social network peer-to-peer influence by adopting new behaviors and messages that promote peer support, racial and sexuality-based pride, and destigmatization of HIV and PrEP. For BSMM, MPowerment model approaches can reduce stigma by fostering a safe environment free from judgment and discrimination, providing greater understanding and acceptance, and improving peer resilience [20]. We hypothesize that this reduction of stigma may lead to more honest discussion of sex and destigmatization of PrEP, both of which may improve PrEP uptake.

Assessing the experiences of BSMM in MPowerment intervention programs is critical to ensuring their efficacy in promoting community support and disseminating HIV prevention messages. There are important challenges related to implementing MPowerment effectively with BSMM. BSMM may enter MPowerment spaces with significant internalized stigma related to race and sexual identity, often originating from family and peers outside of the BSMM community [21, 22]. The internalized stigma may be inadvertently disseminated among group members in adverse ways, stigmatizing other attendees. It may be impossible to completely eliminate such negative peer-to-peer influence even using effective moderation tools (e.g., ground rules to promote constructive conversation). We hypothesize these adverse experiences may negatively affect the efficacy of MPowerment models in several ways. First, stigmatizing experiences hinder participants’ abilities to be vulnerable and trusting within MPowerment intervention spaces, leading to difficulties in honest discussion of sexual risk and self-assessment of potential personal benefits of HIV prevention. Second, stigmatizing experiences may disrupt the development of social connection in these spaces, which is very important for fostering a sense of identity and community with fellow BSMM. The absence of this sense of identity and community can lead to negative self-concept, and internalized homophobia, which is well-documented to be associated with adverse HIV-risk related outcomes among SMM [22–24]. Finally, we hypothesize that participants who experience identity-related stigma at MPowerment events may be less likely to return, resulting in less exposure to positive HIV prevention messaging in the future.

Our study aimed to explore two closely related research questions. First, what are the experiences of BSMM in MPowerment interventions focused on HIV prevention, particularly related to support, stigma, and other potential challenges? Second, how do these experiences facilitate or deter HIV prevention efforts in this population? Our study findings can help inform future peer-based HIV prevention efforts for BSMM, particularly related to destigmatization and social support.

## Methods

### Sample and Recruitment

We recruited 24 participants at 22 MPowerment events in the greater D.C. Metropolitan area (D.C., Maryland, and Northern Virginia) focused on BSMM. Our sample size was sufficient to achieve saturation of themes. After events, attendees were directly approached to assess interest in participating in the study, with a description of study processes and goals, including evaluation of the event attended. Study eligibility was also discussed at this stage; eligibility criteria included being 18 years of age or older, identifying as male, having had a same-sex sexual partner in the past 6 months, identifying as Black or African-American, and having attended an MPowerment intervention event in the past year. All individuals approached for recruitment met all of these criteria. While not pre-specified, all participants were cisgender, had at least one sexual minority identity (e.g., gay, bisexual, same-gender loving, queer), and had a primary residence in the greater D.C. Metropolitan area. Additionally, event attendees were allowed to refer other participants to the study as long as they had attended at least one BSMM-focused MPowerment event in the past year. Participants who showed interest and met eligibility criteria were scheduled for in-depth interviews within the next 7 days and provided an electronic consent form to review in advance.

### Interview Procedures

Each participant completed one in-depth interview via phone. The interviewer discussed the consent form in detail at the start of the interview, answered any questions, and received both written (i.e., electronically signing the consent form via checkbox) and verbal (i.e., participants being asked if they consented to participant in the study, and responding “yes”) informed consent from all participants. Interviews were conducted using a semi-structured interview guide and took 15 to 25 min each. Questions for this study were primarily focused on three specific areas: initial sociodemographics (e.g., “How old are you?”, “What is your sexual identity?”), participant evaluation of the event attended (e.g., “Did you find the event engaging and interesting?”, “What were your interactions with other event attendees like?”, and “Would you come to another event here?”), and HIV prevention communications (e.g., “How comfortable are you discussing HIV with your friends?”, “What do your friends think of PrEP?”). Sociodemographic proportion differences of 15% or more were highlighted for discussion based on the recommendation of BSMM community colleagues. The goal of these interviews was to provide insight into participant perceptions of MPowerment events, experiences within MPowerment event settings, and how HIV prevention messages are communicated among BSMM. All interviews were conducted by the study team lead, who is a member of the BSMM community and experienced in BSMM community–based health service. Participants were compensated $30 for each interview.

### Data Management

All interviews were audio recorded for accuracy and transcribed in a two-step process. First, interviews were transcribed in Descript, an automated transcription service [25]. This converted interviews into editable text which could be utilized in word processing software. Next, a graduate student reviewed transcripts and audio in detail, correcting any errors in the initial automated transcription. Given that the initial automated transcription was approximately 90 to 95% accurate (e.g., only 5 to 10% of words requiring manual changes), this approach was especially efficient. Transcript data was subsequently managed using both Descript and Microsoft Word. A number of precautions were taken to ensure data security and confidentiality, including analysis of audio data only on encrypted, password-protected computers disconnected from public networks, ensuring audio data was never off-site, deletion of audio data from Descript after transcription, and removal of any identifiable information (e.g., names, addresses) from transcripts. All study procedures were approved by the (blinded for peer review) institutional review boards.

### Thematic Analysis

All interview data was analyzed by an analysis team consisting of two faculty members and two graduate students. Two of the team members were also BSMM community members, including the lead researcher. For identifying and describing themes, we utilized inductive data analysis guided by the 6 phases of thematic analysis [26]. First, all four members of the analysis team began by reading and rereading each transcript individually, noting topics of interest and questions (phase 1—becoming familiar with the data). Then, they identified passages and described common themes that occurred (phase 2—generating initial codes). The entire analysis team met biweekly to review and discuss passages and codes that were identified. Next, each of the four analysis team members served as the primary coder for one of the four identified initial codes and independently began reviewing interviews for common themes (phase 3—searching for themes) and categorizing text passages based on themes identified. This was followed by a secondary coding from a different member of the analysis team, allowing for performance of an interrater reliability check to assess agreement between the two assessments for each of the three primary codes. The secondary coder would also code any text that the primary coder may have missed. Any differences between primary and secondary coding were resolved at a team meeting with both coders present. In all instances, the two coders were able to come to an agreement without the intervention of a third coder. After primary and secondary coding was completed, the primary coder reviewed all coded passages and generated thematic key words and phrases that specifically described the main topics of the passages (phase 4—interpreting the themes). The analysis team reviewed these key words/phrases and collectively identified the relatively common key words as main themes and the less common key words as subthemes (phase 5—refining the specifics of the themes). The final list of themes was inductively interpreted by the entire research team consisting of all named authors (phase 6—final analysis) to identify key experiences of BSMM in MPowerment model intervention settings.

## Results

### Sample

The sample consisted of 24 BSMM (Table 1). Among participants, 14 had attended more than one BSMM-focused MPowerment event in the past year, while 10 had only attended one event. Three-quarters of participants were age 25 to 44, and just over half of participants had attended college, with a quarter having a high school diploma as their highest education level. Nearly two-thirds of the sample resided in Maryland, with the rest residing in Washington, D.C. or Virginia. Two-thirds of the sample had never used PrEP, and only a fifth were currently using PrEP. Notably, consistent MPowerment event attendees (e.g., those who attended more than two events in the past year) were more likely to be age 25–34, reside in the Washington, D.C., and were currently using PrEP. First-time attendees were more likely to be younger (age 18–24) and have never used PrEP. We identified four key themes from the transcript analysis process: Black queer intersectional social support and community, social status, HIV-related information and destigmatization, and sexuality. Within each of these themes, we identified relationships with overall HIV prevention messaging and practices. Saturation of themes was achieved within 19 interviews. All quotes are preceded with pseudonyms in quotations, and participant age and attendee status in parentheses (Table 2). Bolded headers are main themes, while subthemes are bolded throughout.

### Black Sexual Minority Intersectional Social Support and Community

The most common theme identified was the sense of intersectional Black sexual minority support and community in MPowerment model settings. The vast majority of participants (83.3%) described MPowerment events as an important source of **connection to other BSMM** and **BSMM social support**. Many participants expressed that this was one of the easiest and most effective ways to make genuine friendships in the BSMM community, including “Devin” (age 18–24, first-time attendee):

“I know this sounds so weird because everyone finds their friends in different ways. Sometimes they find their friends in growing up. Sometimes they find their friends in the club. Sometimes they find their friends in their neighborhoods….But I’ll say organizations that usually center or catered around, you know, like Black gay men. Cause if it’s an area where all of us can get in, then, then it’s gonna be easier to find people like yourself and to find yourself.”

Relating directly to the intersectionality framework, MPowerment events were noted as especially important spaces for **discussion of external intersectional challenges**. “Marcus” described some of the challenges of being an intersectional minority seeking healthcare (age 25–34, multiple-time attendee):

“I would say that trust with the healthcare, already being Black, you know, the Black community already don’t have a great relationship with healthcare. So being gay or queer at the same time, I feel like it is just like, being a double minority especially in a healthcare facility.”

Limitations were noted, however. One challenge that multiple participants mentioned was how social history between participants can lead to stigma within these event spaces. “Antoine” (age 25–34, multiple-time attendee): discusses this in the form of “shade,” referring to stigma and negative judgment within Black queer communities in this context:

“Yes, the event created a welcoming space. And you know, in our (BSMM) community. we talk a lot about shade. And, if you know one person, it’s just one of the unfortunate circumstances. Some history that could be sexual, could be social, could be romantic, it doesn’t matter. But the conditions of that, sort of bubble up. And you can feel it, you can see it, and it creates a context for shade. But in regard to this event, it felt open and it felt like people were self-aware enough to know that even with the history they have with individuals, it was important not to bring that into the space and to color the ways in which our connections were taking shape.”

Finally, participants noted that these spaces often provided an area for discussing HIV and PrEP, as well as **internal intersectional challenges** being Black and a sexual minority in Black spaces. Many participants discussed the clear link between the ability for BSMM to discuss their intersectional experiences and engagement with HIV prevention, including “Jamal” (age 35–44, multiple-time attendee):

“It gives Black men who have sex with men opportunities to talk about PrEP, to talk about relationships, to talk about HIV, just talk about our daily lives and things that we sort of consider to be opportunities for connection, networking and our self-esteem, self-image…And so, for example, the Black church, in Black communities, in Black masculinity, (we) aren’t necessarily fitting into those concepts from those communities that we identify with (but) not necessarily being able to align with them. And then being able to come together and recognize the ways in which those unfavorable societal responses has contributed to a poor self-esteem or a poor self-concept, and moving with that as an opportunity to recognize that we could lean on one another and that we could provide each other safe support, safe spaces.”

### Social Presentation

Social presentation was a frequently reported (66.7%) to be a barrier to engagement with MPowerment events, both in terms of entry to events and engagement with event activities. Participants often described this in contexts unique to the D.C. Metropolitan area, such as having a “government job,” which was discussed as providing greater **socioeconomic presentation** and a sense of social distance from other BSMM. “Deonte” (age 18–24, multiple-time attendee) noted challenges with getting BSMM community members with a high self-perceived social status to engage in event activities:

“I’m noticing there’s this sort of like, for lack of (a) better term, upper echelon group of folks in this area specifically that might make a little bit more money, might have government jobs, or might feel like they’re too good to be associated with other groups of people within our communities.”

Social standards around **body image** were also described as challenges in some MPowerment event spaces, particularly those based in bar and club venues, where expectations around physical and socioeconomic presentation may be heightened. While many participants noted enjoyment with health activities taking place in a less clinical setting, a commonly identified challenge was requirements around this presentation in these spaces, especially related to body image and income. “Shaun” (age 25–34, first-time attendee) and “Jalen” (age 35–44, first-time attendee) described this as defeating the purpose of MPowerment spaces as inclusive and safe, yet being inevitable in certain venue spaces:

“Sometimes you’re just not the image of what a gay man should look like or a gay Black man should look like in a sense. So, trying to conform to these standards, but also trying to still be yourself is so hard….I experienced a lot of ‘Oh, you don’t have this’ or ‘you don’t have that,’ and I don’t think this space is supposed to be about that.”

“You know, having the perfect chiseled bodies or, you know, being over six foot. It’s a lot of things that comes with the territory, but um, those are the things that you kind of have to live up to. Be able to make over six figures (and) have the latest and greatest outfits and designers. So that’s pretty much the image that they, well, our community usually go for, and some of the spaces, you know, usually cater to.”

The discussion of what a gay Black man should look like highlights the relevance of intersectionality to **general presentation**. Intersectional challenges can include within-group body image standards, as well as socioeconomic expectations and difficulties. These challenges were described as deterring effective HIV prevention messaging. If the community views a particular space as having significant requirements based on body type, income, and general presentation, it becomes a substantial barrier to engagement. “Jalen” further described the challenges in MPowerment event health promotion given stigma and exclusion related to social status:

“But for real, you know how it is. They think that because they got a little money and a nice apartment they don’t really need, like, they don’t need you. And the end of it, how are you gonna get them to take PrEP or get tested if they think they’re better than you?”

### HIV-Related Information and Destigmatization

The majority of participants (79.1%) discussed MPowerment events as an important source of both **HIV and PrEP knowledge**. “Shaun” (age 25–34, first-time attendee) gave this response when asked “do you think that the topics discussed at the event were relevant to you and relevant to your community?”:

“Oh, absolutely. It’s relevant because there’s so many who really don’t know that there is HIV for bridge, HIV prevention, medication known as PrEP, and, and that this is, um, medication that is accessible to everyone…So this is vital because it helps break the stigma around HIV.”

**Destigmatizing both HIV and PrEP** was described as an effective way to get participants engaged with PrEP. Participants also appreciated integration of HIV prevention messaging with overall wellness, health, and sexuality. A commonly mentioned barrier to larger HIV prevention engagement was focusing too heavily and aggressively on HIV/STI prevention without a context of overall wellness. MPowerment events were generally praised for avoiding this pitfall, such as by “Deonte” (age 18–24, multiple-time attendee):

“It was mostly, um, education about HIV prevention and how STIs can be transmitted. But then also tips on how to have like, you know, a better sex life…It was definitely a mixture of like health and wellness mixed with, um, STI prevention. So it wasn’t so much like STI prevention like shoved down your face.”

Finally, participants noted how messaging in these spaces helped promote HIV prevention through **destigmatizing HIV and PrEP**. This created more comfort asking questions regarding PrEP and using it regularly. “Corey” (age 25–34, multiple-time attendee) who was introduced to PrEP through a MPowerment event described this:

“And so now just having that same sense of like, pride or sense of security has kind of carried through me being able to receive more information and get better educated on the issues related to HIV, including stigma. To kind of realize that it’s okay to just talk about these issues and not feel any shame or judgment. Regardless of what people think about me, because I know that I’m doing what I feel is the best for my health.”

### Sexuality

The majority of participants (79.1%) discussed promoting openness and comfort around sexuality as one of the most important experiences in MPowerment events. “Amari” (35–44, first-time attendee) noted that **supporting sexuality** was very important to encouraging open discussion:

“So the leader of the session was a sex expert that had like done some studying of various philosophies related to sexual health and wellness. And so, I think the environment, like just him being a presence in that room set this tone of freedom of self-expression as it relates to sexuality.”

Some participants reported a **need for greater inclusion of sexual identities** in how MPowerment programs are promoted. “Javan” (45–49, first-time attendee), described difficulties related to inclusion of the full spectrum of sexual minority identities:

“They should be inclusive of, you know, the broad range of sexual identities. It’s not just, you know, gay, bi, queer, you know, and so forth. I’ve wanted to go to (an) event but didn’t feel comfortable because gay was the only thing in the description. But if you put this identifier very high up, then I’m automatically like, oh, that’s not for me kind of situation. Um, whereas if we talk about an organization being a safe space for men who at some point in time, have had sex with men, it’s different. We (organizations) should be very clear with how we name oursel(ves), how we define oursel(ves), so that when persons come to our website or come to our social media pages, they have a clear understanding that we support the full stratification of sexuality.”

Finally, participants discussed how **destigmatizing sex** is important to people accepting HIV prevention messaging. “Antoine” (25–34, multiple-time attendee), a long-time event attendee, describes the importance of destigmatizing sexuality in getting BSMM to be open to prevention and treatment:

“I’m someone who wants to get rid of the stigma around sexuality, cause there’s a stigma with that too, where sex was something that was dirty and you didn’t talk about it. You kind of kept it, hush, hush, you know, and a lot of us come from, you know, generations where that negative way of thinking has kind of been passed on , but it’s like sex is not dirty. It’s only based on your actions of how you handle sex, but openly talking about it and being honest about your sexual desires is actually groundbreaking. And it actually helps you to be open to what’s available as far as your (HIV) prevention, your treatment, and also your understanding of how sexuality, what sexuality really is.”

## Discussion

Consistent with much of the literature, MPowerment event spaces provide a forum for BSMM to feel safe and supported in discussing many of the intersectional challenges they face [20, 27–29]. The intersectional component is critical, as participants frequently noted these spaces were a venue to discuss difficulties related to both race and sexuality. This also provided a means for BSMM to connect to one another; this is especially important given the well-documented challenges queer people face related to social isolation and its related adverse health outcomes (e.g., depression, anxiety, substance use) [30–32]. In the context of HIV prevention, many participants described more ease in accessing these services through community-based MPowerment interventions compared to more general healthcare settings. The supportive and intersectionally understanding environment of MPowerment model programs was described as exceptionally helpful for delivering HIV prevention messaging in an effective way, and as a means of helping address many of the challenges BSMM face [20]. This was also reflected in our descriptive findings, where repeat attendees were more likely to utilize PrEP than first-time attendees.

Participants also noted elements of social presentation fairly unique to the D.C. metropolitan area, such as social value related to having a government job. This was described as most critical to events related to bar and club settings, where there are greater cultural expectations around socioeconomic presentation and general presentation and appearance. This is especially important given that participants noted these settings were otherwise desirable as event locations given that they are entertainment-focused, inherently social, and generally well known in SMM communities. While these are useful advantages, the focus on presentation in club and bar settings is often a significant barrier to entry for much of the BSMM community. More broadly, this also highlights the importance of regional cultural context in developing HIV prevention promotion events. BSMM culture is diverse across many different regional and state contexts, and this diversity should be reflected in the approach to HIV prevention messaging. Training for event facilitators should include both an understanding of the nuanced forms of stigma, as well as the venues available to BSMM, and general norms related to engaging with members of the community. This may underscore elements of power ascribed to social status within BSMM communities. Further investigation is needed to understand how this form of power influences behavior around the use of PrEP in BSMM relationships.

Overall, efforts to destigmatize PrEP uptake are essential to reduce the acquisition of HIV and effectively curb existing trends; this is a critical function of MPowerment interventions with implications for the HIV epidemic [2–7]. Amid major advancement in HIV treatment and biomedical prevention strategies such as PrEP, there is compelling evidence that misinformation and stigma continue to impact rates of uptake and PrEP adherence among BSMM [33]. PrEP usage is often stigmatized as contrasting to abstinence and condom usage historically promoted as HIV risk reduction strategies [34]. Stigma of this nature further stymies efforts to end the HIV epidemic and exacerbates assumptions that individuals actively using PrEP are tainted, devalued, and thus stereotyped as having uncontrollable sexual behaviors [34]. Similarly, sexual stigma is a well-documented barrier to HIV prevention overall, in part due to deterring honest discussions around sex and sexual health. [34–36]

When developing MPowerment model interventions, there are several important factors to consider drawn from our findings. Destigmatizing sexuality may significantly improve event attendee comfort level in discussing their sexual identities, experiences, and beliefs. This facilitates more effective PrEP promotion and consideration of additional HIV prevention methods. The organizations and individuals leading MPowerment events for BSMM play an important role in creating a space where attendees feel accepted, understood, and supported. Providing a space that actively encourages peer social support can be critical to encouraging BSMM to accept their identities. Inclusion for all BSMM irrespective of socioeconomic status and presentation is especially important. These are all considerations to maximize the success of MPowerment-based HIV prevention for BSMM.

There are important strengths to our research to consider. We were able to identify key thematic patterns that directly related to HIV prevention efforts, helping elucidate key relationships between MPowerment model events and HIV prevention. These can translate into direct actionable items for developing peer-based HIV prevention events, such as through facilitator training. Participants noted a strong rapport with the interviewers and a genuine interest in the research, in part because the research lead (who was also the interviewer) is a member of the BSMM community well-connected to BSMM community–serving organizations. Finally, our study helps fill a gap in the literature on BSMM experiences in MPowerment model settings, particularly in the D.C. metropolitan area.

This study is not without limitations, however. We focused on BSMM in the D.C. Metropolitan area who had attended at least one MPowerment event. Thus, our results may not reflect experiences of other BSMM populations in different geographical locations, SMM of other racial/ethnic groups, or BSMM who have never attended MPowerment events. The focus on HIV prevention among BSMM is scientifically grounded however given their greater vulnerability to HIV acquisition, and thus the greater importance of HIV prevention efforts. Similarly, the D.C. metropolitan area is a focal point of HIV prevention efforts given the greater regional HIV prevalence. Differences in the topics, attendees, and design of MPowerment events may affect participant responses and generated themes. While the sample size is relatively small, we were able to reach saturation of themes and gain a very detailed understanding of participant experiences. Finally, the sensitive nature of many of the topics, particularly related to sex, sexuality, and experience stigma, is likely to be affected by social desirability bias. Despite this limitation, participants reported many vulnerable and stigmatized experiences, in part due to the aforementioned trusting rapport with the interviewer.

## Conclusion

Overall, MPowerment event spaces provide a forum for BSMM to feel safe and supported while gaining important HIV-related knowledge and prevention access. We found that Black sexual minority intersectional social support and community, social status, HIV-related information and destigmatization, and sexuality were key themes of BSMM experiences in MPowerment model settings. Each of these themes has significant implications for HIV prevention efforts towards this population, particularly related to PrEP utilization. Regional cultural factors, particularly related to the context of social status in the greater D.C. metropolitan area, were also noted as relevant to engagement with MPowerment event spaces. Each of these factors are important considerations in the design and implementation of these events, as well as similar HIV prevention programming efforts towards BSMM. Future quantitative studies exploring experiences of BSMM in these events, with a focus on several dimensions of stigma, are recommended. Additionally, further research into BSMM who do not engage with MPowerment programming, and the specific barriers to engagement, can better inform health promotion outreach efforts to this community.

## Figures and Tables

**Table 1:** Characteristics of interviewed Black sexual minority men in MPowerment intervention events (*n* = 24)

	Total (*n* = 24)	Multiple-time event attendees^1^ (*n* = 14)	One-time event attendees^1^ (*n* = 10)
**Age**			
18–24	20.8%	**14.3%**	**30.0%**
25–34	37.5%	**50.0%**	**20.0%**
35–44	37.5%	42.9%	30.0%
45–49	8.3%	**0.0%**	**20.0%**
**Ethnicity**			
Non-Hispanic/Latino	83.3%	85.7%	80.0%
Hispanic/Latino	16.7%	14.3%	20.0%
**Highest completed education level**			
High school	25.0%	21.4%	30.0%
Undergraduate college	58.3%	57.1%	60.0%
Graduate college	16.7%	21.4%	10.0%
**State of residence**			
District of Columbia	20.8%	**28.6%**	**10.0%**
Maryland	62.5%	57.1%	70.0%
Virginia	16.7%	14.3%	20.0%
**PrEP use**			
Never	66.7%	**57.1%**	**80.0%**
Previous use	12.5%	14.3%	10.0%
Current use	20.8%	**28.6%**	**10.0%**

“One-time event attendees” includes participants who only attended one event in the past year. “Consistent event attendees” includes participants who have attended more than two events held by Black sexual minority men’s community–based organizations in the past year (no participants attended exactly two events). Proportion differences > 15% are bolded

**Table 2:** Themes, subthemes, and example quotes from interviews with Black sexual minority men in MPowerment intervention events (*n* = 24)

**Black sexual minority intersectional social support and community**	
** Connection to other BSMM (83.3%)**	“*Everyone finds their friends in different ways. Sometimes they find their friends in growing up. Sometimes they find their friends in the club. Sometimes they find their friends in their neighborhoods….But I’ll say organizations that usually center or catered around, you know, like Black gay men. Cause if it’s an area where all of us can get in, then, then it’s gonna be easier to find people like yourself and to find yourself*.”
** BSMM social support (75.0%)**	“*It gives Black men who have sex with men opportunities to talk about PrEP, to talk about relationships, to talk about HIV, just talk about our daily lives and things that we sort of consider to be opportunities for connection, networking and our self-esteem*.”
** Discussion of external intersectional challenges (66.7%)**	“Y*ou know, the Black community already don’t have a great relationship with healthcare. So being gay or queer at the same time, I feel like it is just like, being a double minority especially in a healthcare facility*.”
** Internal intersectional challenges (58.3%)**	“*I love my brothers (BSMM), but baby we make it hard for each other. There’s a lot of love between us but there’s a whole lotta hate too*.”
**Social presentation**	
** Socioeconomic presentation (66.7%)**	*“There’s this sort of like, for lack of (a) better term, upper echelon group of folks in this area specifically that might make a little bit more money, might have government jobs, or might feel like they’re too good to be associated with other groups of people within our communities*.”
** General presentation (50.0%)**	***“****Sometimes you’re just not the image of what a gay man should look like or a gay Black man should look like in a sense. So, trying to conform to these standards, but also trying to still be yourself is so hard*.”
** Body image (29.2%)**	***“****You know, having the perfect chiseled bodies or being over six foot. It’s a lot of things that comes with the territory, but um, those are the things that you kind of have to live up to*.”
**HIV-related information and destigmatization**	
** HIV knowledge (79.1%)**	“*It was mostly, um, education about HIV prevention and how STIs can be transmitted. But then also tips on how to have like, you know, a better sex life…It was definitely a mixture of like health and wellness mixed with, um, STI prevention*.”
** HIV destigmatization (66.7%)**	“*And so now just having that same sense of like, pride or sense of security has kind of carried through me being able to receive more information and get better educated on the issues related to HIV, including stigma. To kind of realize that it’s okay to just talk about these issues and not feel any shame or judgment*.”
** PrEP knowledge (66.7%)**	***“****It’s relevant because there’s so many who really don’t know that there is HIV for bridge, HIV prevention, medication known as PrEP, and, and that this is, um, medication that is accessible to everyone*.”
** PrEP destigmatization (54.2%)**	“*And there’s so much stigma around both HIV, AIDS, and PrEP still. So, it is important for me as a person who was able to access it based on privileges and knowledge and access (to MPowerment spaces), that I’m able to share this information with others*.”
**Sexuality**	
** Supporting sexuality (79.1%)**	*“One thing they (MPowerment spaces) can do is have more town hall meetings, or like support groups, where people can create a safe space where people can actually express their sexual identities, their gender identities, their sexual experiences*.”
** Destigmatizing sex (62.5%)**	“*I’m someone who wants to get rid of the stigma around sexuality, cause there’s a stigma with that too, where sex was something that was dirty and you didn’t talk about it*.”
** Need for greater inclusion of sexual identities (25.0%)**	“*They should be inclusive of, you know, the broad range of sexual identities. It’s not just, you know, gay, bi, queer, you know, and so forth*.”

Parentheses after each subtheme contain the percentage of participants interviews where that theme was observed

## Data Availability

Please contact Rodman Turpin (rturpin@gmu.edu) for data requests.
